# An automated proteomic data analysis workflow for mass spectrometry

**DOI:** 10.1186/1471-2105-10-S11-S17

**Published:** 2009-10-08

**Authors:** Ken Pendarvis, Ranjit Kumar, Shane C Burgess, Bindu Nanduri

**Affiliations:** 1 Institute for Digital Biology, Mississippi State University, Mississippi State, MS 39762, USA; 2 College of Veterinary Medicine, Mississippi State University, Mississippi State, MS 39762, USA; 3 Mississippi Agriculture and Forestry Experiment Station, Mississippi State University, Mississippi State, MS 39762, USA; 4 MSU Life Sciences and Biotechnology Institute, Mississippi State University, Mississippi State, MS 39762, USA

## Abstract

**Background:**

Mass spectrometry-based protein identification methods are fundamental to proteomics. Biological experiments are usually performed in replicates and proteomic analyses generate huge datasets which need to be integrated and quantitatively analyzed. The Sequest™ search algorithm is a commonly used algorithm for identifying peptides and proteins from two dimensional liquid chromatography electrospray ionization tandem mass spectrometry (2-D LC ESI MS^2^) data. A number of proteomic pipelines that facilitate high throughput 'post data acquisition analysis' are described in the literature. However, these pipelines need to be updated to accommodate the rapidly evolving data analysis methods. Here, we describe a proteomic data analysis pipeline that specifically addresses two main issues pertinent to protein identification and differential expression analysis: 1) estimation of the probability of peptide and protein identifications and 2) non-parametric statistics for protein differential expression analysis. Our proteomic analysis workflow analyzes replicate datasets from a single experimental paradigm to generate a list of identified proteins with their probabilities and significant changes in protein expression using parametric and non-parametric statistics.

**Results:**

The input for our workflow is Bioworks™ 3.2 Sequest (or a later version, including cluster) output in XML format. We use a decoy database approach to assign probability to peptide identifications. The user has the option to select "quality thresholds" on peptide identifications based on the P value. We also estimate probability for protein identification. Proteins identified with peptides at a user-specified threshold value from biological experiments are grouped as either control or treatment for further analysis in ProtQuant. ProtQuant utilizes a parametric (ANOVA) method, for calculating differences in protein expression based on the quantitative measure ΣXcorr. Alternatively ProtQuant output can be further processed using non-parametric Monte-Carlo resampling statistics to calculate P values for differential expression. Correction for multiple testing of ANOVA and resampling P values is done using Benjamini and Hochberg's method. The results of these statistical analyses are then combined into a single output file containing a comprehensive protein list with probabilities and differential expression analysis, associated P values, and resampling statistics.

**Conclusion:**

For biologists carrying out proteomics by mass spectrometry, our workflow facilitates automated, easy to use analyses of Bioworks (3.2 or later versions) data. All the methods used in the workflow are peer-reviewed and as such the results of our workflow are compliant with proteomic data submission guidelines to public proteomic data repositories including PRIDE. Our workflow is a necessary intermediate step that is required to link proteomics data to biological knowledge for generating testable hypotheses.

## Introduction

Recent advances in genome sequencing projects have facilitated the global analysis of proteins ("proteomics") in order to study their role in health and disease. Proteomic datasets may be generated by coupling nanoflow technology with high-speed, high resolution mass spectrometers and these have generated immensely complex and very large mass spectral datasets. Analyzing these huge datasets by hand is a daunting, inefficient, and error-prone task, hence the need for an automated data analysis pipelines.

Multidimensional Protein Identification Technology (MudPIT) [1] followed by database searching is commonly used to identify proteins from a biological sample. Biological problems addressed by proteomics often include comparing two different conditions, e.g. normal versus treatment. For comparative proteomics, there is a need to determine which subset of proteins is differentially expressed (DE) at a defined statistical threshold. Sample preparation for proteomics includes total protein isolation from a target biological sample and digestion of these proteins using proteases like trypsin to generate a complex mixture of peptides that then need to be deconvoluted and analyzed by mass spectrometry. One method to reduce the complexity of peptides is separation based on their charge and hydrophobicity using two-dimensional liquid chromatography (2D-LC) before the peptides enter the mass spectrometer for MS/MS analysis.

The flow rates required to separate peptides are in the nanoliter to microliter per minute range and mass spectrometers must collect data for an extended amount of time, often for many hours. The resulting data sets can contain 10s to hundreds of thousands of mass spectra, which must then be searched against a protein database to identify the peptides and thus the proteins. The protein database is in silico digested with a protease (used for sample preparation) to generate database of peptides and their theoretical spectra that can be matched with the experimental spectra collected by mass spectrometry. Several search algorithms are described in literature for database searching, including Sequest [2], MASCOT [3], and X!Tandem [4] which match experimental mass spectra to theoretical spectra derived from a protein database. Sequest is a widely used searched algorithm and our proteomics workflow is designed to analyze Sequest search results. Sequest computes a cross correlation (Xcorr) function to assess the quality of peptide spectra matches. The better the match between an experimental peptide mass spectrum and its database counterpart, the higher the Xcorr will be. Sequest also computes ΔCn, a normalized score calculated from XCorr difference between the best peptide match and the second best match. ΔCn is dependent on database size, search parameters, and sequence homologies. While both XCorr and ΔCn have been used widely in the past for filtering search results [5-8] they provide little information for distinguishing correct peptide assignments from false positives. To get the most meaningful biological data from proteomics or any high throughput experiment it is necessary to reduce the false discovery rate. Decoy database search methods for estimating probabilities for peptide identifications are described in literature [9,10]. However, open source computational tools that automate this estimation are not readily available.

Beyond the identification of peptides and proteins at acceptable statistical thresholds, for expression proteomics the end user requires computational tools for differential protein expression. Label free protein quantification methods determine relative protein abundances directly from high throughput proteomic analyses with out labeling techniques using sampling statistics like spectral counting [11], number of peptides [12], and ΣXcorr [13]. We developed ProtQuant, a java based tool for label free quantification that uses a spectral counting method with increased specificity based on ΣXCorr. However, ProtQuant computes the statistical significance of differential protein expression using parametric statistics (ANOVA) assuming that the distribution of the control and treatment datasets closely approximates a normal distribution. However, this assumption may not be valid for shotgun proteomics due to either the biology under investigation or due to small sample sizes common to proteomic studies resulting in type I errors (i.e. increased false positive significance rate). Computer intensive distribution-free statistics offer a solution to this problem and we have applied random resampling with replacement to determine statistically significant differences in protein expression from ESI MS^2 ^data [14].

A recurring theme in high-throughput biology is that collecting orthogonal evidence for biology under investigation using complementary data analysis platforms could reduce the noise and identify true biological effects. For example, microarray differential expression analysis is often complemented by quantitative RT-PCR. Matching mass spectra using two different algorithms like Sequest and Mascot often generates a list of proteins that overlap but also proteins uniquely identified by each method. Likewise given enough computational resources and automated data analysis tools, biologists could evaluate differential protein expression using different statistical tests to identify a core set of differences that could represent true biological changes in expression. Furthermore, proteomic analysis workflows also require corrections for multiple testing to reduce false positive identifications of significant DE based on a single P value cutoff.

**Figure 1:**
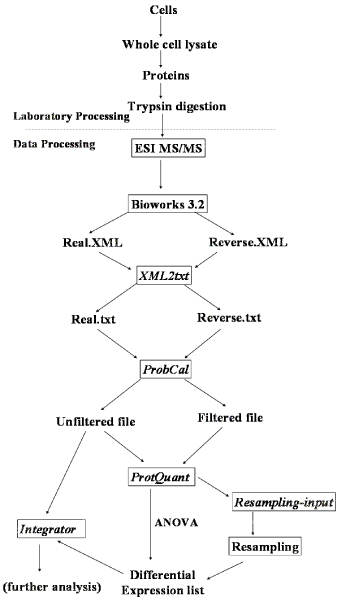
**Proteomics data analysis workflow**.

Here we describe a computational pipeline that automates the data analysis workflow from assigning probabilities for peptide identification using decoy database approach to statistical evaluation of protein DE using ANOVA and resampling statistics, with subsequent correction for multiple testing using Benjamini and Hochberg's method [15]. This integrated work flow (Figure 1) combines some of our open source software tools like ProtQuant and additional scripts to generate an output that has a list of proteins from a biological sample together with peptide and protein probabilities. Where the experimental design includes comparative proteomics, P values for protein DE adjusted for multiple testing are given for ANOVA based and resampling based (optional) methods.

To illustrate the functionality of our proteomics workflow we used the *Edwardsiella ictaluri *response to iron restriction using 2,2-dipyridyl (DP) iron chelator. *E. ictaluri *cultures were grown in triplicate and outer membrane proteins were isolated. Mass spectrometry and Sequest searches with a protein and reversed-protein database were done as previously described [14]. SEQUEST results were processed using the tools and scripts described in our workflow.

## Results

Our proteomics workflow starts with SEQUEST search results in XML format from Bioworks 3.2 browser for both protein and reverse database searches. We chose the XML format as a standard format for Bioworks output as it overcomes the 65536 row file size limit for some versions of Microsoft Excel spreadsheets. When exporting Bioworks 3.2 search results, we recommend that the user does not apply any filters. However, due to the virtual memory constraints imposed by computer desktops, if exporting without filters is not practical, we suggest applying minimal filters for peptide charge state. However, the end users need to be aware that if the peptide filters are set too high, many positive matches may be lost. We created a java script named XML2TXT to quickly convert the XML output files to tab delimited text files, which are used by other scripts and can be opened in Excel/notepad for viewing.

Once the real and reverse unfiltered data files are formatted properly using XML2TXT, they can be processed by ProbCal. ProbCal is a set of PERLscripts that automate the estimation of peptide probabilities using search results from a protein and a decoy database. A t-score is obtained for each Xcorr and ΔCn pair from the reverse search results and based on this score a P value is calculated for peptides identified from protein database. The results can then be filtered using a probability cutoff, typically p ≤ 0.05.

**Figure 2:**
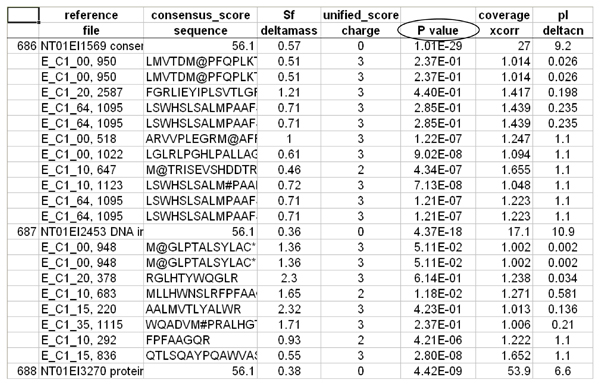
**Sample ProbCal output showing protein and peptide probabilities**.

**Figure 3:**
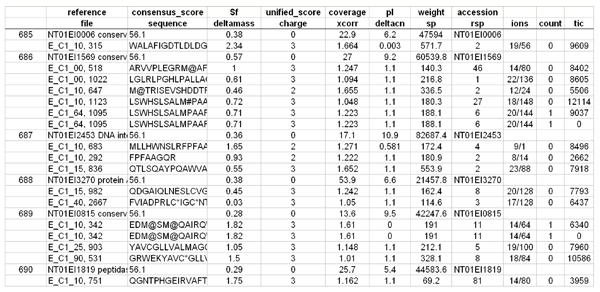
***E. ictaluri *data after being filtered by ProbCal-filter with P < 0.05**.

Individual peptide probabilities are further utilized to calculate protein probability using published methods [16]. Another subsidiary script ProbCal-filter uses the peptide probabilities to filter low quality peptides from being included in further analysis. ProbCal can be run from the command prompt, with the names of the real and reverse databases as arguments. For each real/reverse dataset pair a single tab-delimited text file is created with a column containing calculated probabilities for each protein and its associated peptides (Figure 2). We used ProbCal and ProbCal-filter to filter our *E. ictaluri *data with a peptide P value cutoff for protein identification of <0.05 (Figure 3). If differential expression is not the goal of the researcher, then the analysis is complete after ProbCal, otherwise the data is now ready to be processed by ProtQuant. Processing *E. ictaluri *datasets with ProbCal identified 3482 proteins from the normal growth condition (iron replete) and 3437 proteins from growth in the presence of iron-chelator DP at P ≤ 0.05 for peptide probability. The probability of a protein identification being incorrect was ≤ 0.030 for all identified proteins in the control dataset and 0.038 for proteins identified in DP dataset.

**Figure 4:**
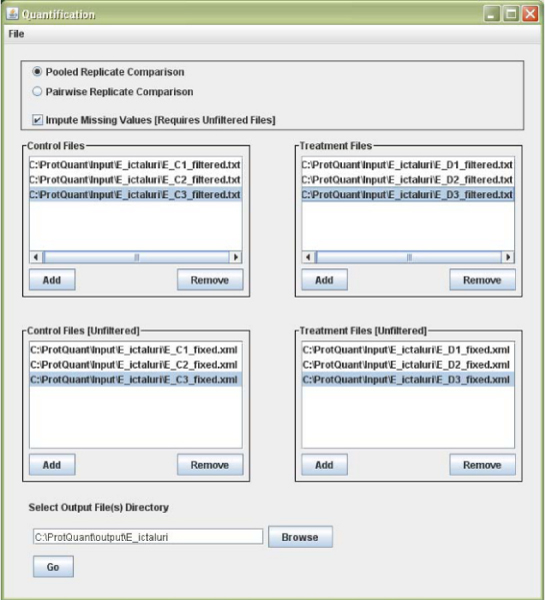
**ProtQuant graphical user interface**.

The next step in our proteomics workflow after filtering the initial search results is ProtQuant. ProtQuant, written in Java, is installed using a self extracting executable file downloadable form our AgBase website http://agbase.msstate.edu/. ProtQuant has a graphical user interface (Figure 4) for choosing control and treatment files and accepts the output files directly from ProbCal to perform DE analysis by ANOVA. To fill in the "missing Xcorr values", ProtQuant also requires the corresponding original unfiltered Sequest XML output for each dataset that is analyzed [13]. The built-in XML conversion tool in ProtQuant converts XML files to .txt files.

**Figure 5:**
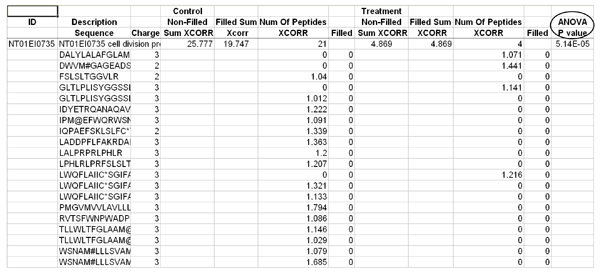
**Sample ProtQuant output**.

ProtQuant has several modes of operation; it can analyze replicates as pairs or as pooled replicates and generate a single output file. A simple check-box can be selected to activate the function to fill in missing Xcorr values. Once the appropriate input files are selected as either controls or treatments and the output directory is specified, clicking "Go" will start the differential expression analysis. We chose to analyze our *E. ictaluri *results from ProbCal in ProtQuant as control (iron replete) versus iron restricted (DP). The output from ProtQuant is a text file containing a list of proteins present in the combined replicates for control and treatment datasets with an ANOVA P value in the last column for protein DE (Figure 5). ProtQuant computes the statistical significance of DE for proteins using one-way ANOVA. This method requires at least 3 peptides for each protein from the combination of the control and treatment to calculate P value. Using a custom Perl script process_ProtQuant we do the correction for multiple testing based on the published method of Benjamini and Hochberg. Another perl script add_protein_prob combines ProbCal and ProtQuant outputs to generate a master output file with proteins identified from control and treatment datasets and also additional information for each protein including: numbers of peptides used for protein identification, ΣXcorr, peptide probability and protein probability, ANOVA P value, and significance after multiple testing correction.

ProtQuant analysis of the *E. ictaluri *iron replete and DP comparison identified expression of 217 proteins to be significantly increased or decreased (at Benjamini-Hochberg adjusted p ≤ 0.05).

**Figure 6:**
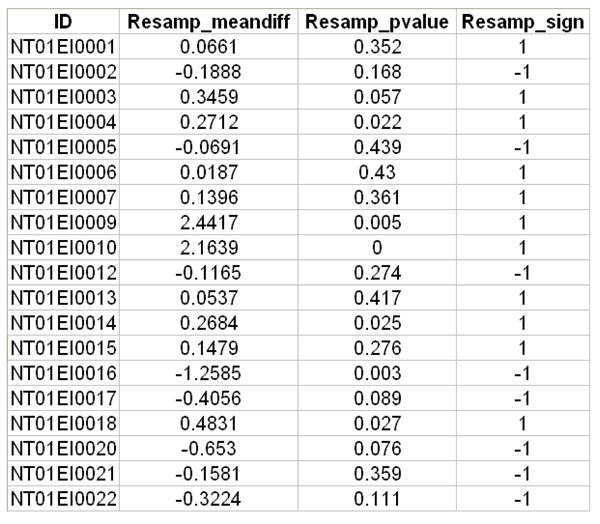
**Sample resampling output generated by rsProt**.

Since ProtQuant output has the compiled information for all replicate datasets for two conditions that are being compared, we use ProtQuant output as a template for performing resampling-statistics-based DE analysis using our MATLAB script, rsProt. The first step is to reformat the ProtQuant output to remove protein entries that do not have at least three peptides in at least one dataset that is being compared using a Perl script, resampling-input. The output of resampling-input is in the required format for running rsProt in MATLAB. To run rsProt, the first line in rsProt must be modified to match the filename of the sample to be analyzed by resampling and the last line can be modified only if a specific output file name is desired. rsPRot requires a user specified number of iterations for estimating the P value. After running rsProt a text file is generated containing four columns (Figure 6). Columns one, two, three, and four contain the protein id, mean difference in the ΣXcorr, probability, and direction of differential expression relative to the control dataset, respectively (1 represents increased expression of a protein in the treatment compared to the control and -1 represents opposite trend).

**Figure 7:**
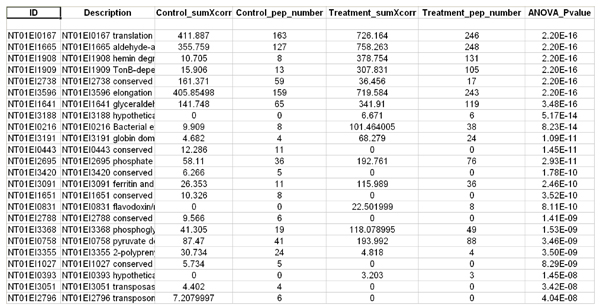
**Combined output from integrator showing ProtQuant data**.

**Figure 8:**
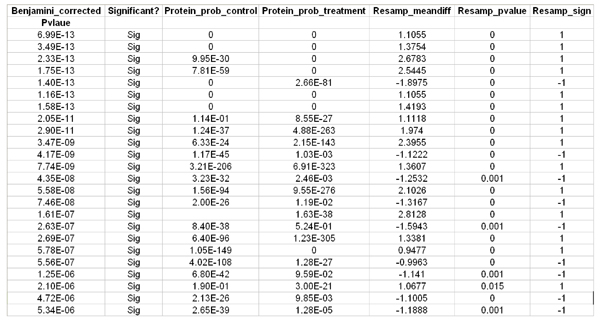
**Combined output from integrator showing Benjamini-Hochberg correction, protein probabilities and resampling data**.

The final step in our proteomics workflow that includes resampling statistics is to compile the results from ProbCal, ProtQuant, and rsProt to generate a comprehensive list of differentially expressed proteins. A collection of custom Perl scripts entitled integrator is used to accomplish this task. Integrator is run in steps from the command prompt and produces a single text file. Step one is to run process-protquant which reduces the ProtQuant data to a list of significantly expressed proteins with ΣXcorr for the control and treatment and performs a Benjamini-Hochberg correction. Step two is to run add_protein_prob which adds the protein probabilities calculated by ProbCal to the ProtQuant results. The final step is to run add_resampling_data which adds the columns from the resampling results to the file. The compiled results contains a master list of differentially expressed proteins, the P values calculated by ProbCal indicating confidence in identification, relative expression data from ProtQuant, and resampling data indicating the probability of being wrong that the protein is differentially expressed. Figures 7 and 8 show a sample of the *E. ictaluri *data after being compiled by integrator.

## Conclusion

The proteomic data analysis workflow described here for Bioworks Sequest results includes a modular design of the work flow wherein different components can be combined together to perform different analyses. The work flow can be as simple as identifying proteins at a certain probability threshold or as extensive as comparing two datasets for differential protein expression using multiple statistical methods. All the tools and scripts described here can be implemented and further modified to accommodate additional analyses design but do require basic programming skills. All the tools and scripts used are compatible with both Linux and Windows platforms.

## Methods

### Implementation

XML2TXT is a java program that converts an xml file into a tab delimited text file, further used by other scripts. It is implemented using Xalan-Java, which is an XSLT (XSL Transformations) processor for transforming XML documents into text document types. javax.xml.transform interface is used as java API for XML Processing (JAXP) 1.3.

Perl scripts ProbCal, ProbCal-filter and integrator require the installation of the Active Perl runtime environment available at http://www.activestate.com/activeperl/. ProbCal is the implementation of the peptide probability calculation. Individual peptide probabilities are further utilized to calculate the probability that a protein identification is incorrect. Another subsidiary script ProbCal-filter uses the peptide probabilities to filter low quality peptides from being included in further analysis.

ProtQuant is implemented in Java 5 for platform independence. A self-installing executable for Windows has been generated using Macrovision InstallShield. An instruction for installing and using the tool in a Linux environment is also available. ANOVA analysis is done using a library from the R statistical package http://www.rproject.org/. Because of the size of the datasets that ProtQuant must handle, MySQL is used for data storage and efficient data manipulation. ProtQuant uses the file extension of input files to determine the format. ProtQuant includes a custom built parser for XML files. rsProt, for resampling, requires the installation of MatLab, available for purchase from MathWorks at http://www.mathworks.com/products/matlab/.

### *E. ictaluri *Proteomics

*E. ictaluri *cultures were grown in triplicate in BHI (iron replete) and BHI with 100 M dipyridyl (iron restriction). Outer membrane proteins were isolated by sodium N-lauroylsarcosinate (SLS) extraction [17]. Protein concentrations were determined using the Plus one 2D quant kit following the manufacturer's protocol (Amersham Biosciences, Piscataway, NJ). Trypsin digestion proteins and analysis of tryptic peptides by 2-D LC ESI MS/MS were conducted as described previously [14]. For protein identification all searches were done using TurboSEQUEST™ (Bioworks Browser 3.2, ThermoElectron). Mass spectra and tandem mass spectra were searched against an in silico trypsin-digested *E. ictaluri *protein database (3786 proteins). Cysteine carboxyamidomethylation and methionine single and double oxidation were included in the search criteria. For decoy searches a reversed version of the protein database was generated using the reverse database function in Bioworks 3.2. The reversed database was also in silico trypsin digested and used for searches with tandem mass spectra exactly as described for the protein database. Bioworks results were exported in XML format for proteomic analysis workflow described here.

## Competing interests

The authors declare that they have no competing interests.

## Authors' contributions

BN and SCB developed/implemented the methods for proteomic analysis. RK wrote the scripts for joining different components of the workflow. KP conducted proteomic data analysis of *E. ictaluri *raw mass spec data (including Bioworks 3.2 searches) and did extensive testing of the analysis workflow. KP wrote the draft of the manuscript. All authors contributed to writing the manuscript and have read and approved the final manuscript.
